# A Real-Time Global Re-Localization Framework for a 3D LiDAR-Based Navigation System

**DOI:** 10.3390/s24196288

**Published:** 2024-09-28

**Authors:** Ziqi Chai, Chao Liu, Zhenhua Xiong

**Affiliations:** 1 State Key Laboratory of Mechanical System and Vibration, School of Mechanical Engineering, Shanghai Jiao Tong University, Shanghai 200240, China; chaiziqi@sjtu.edu.cn (Z.C.); aalon@sjtu.edu.cn (C.L.); 2 Hai’an Institute of Intelligent Equipment, Shanghai Jiao Tong University, Nantong 226600, China

**Keywords:** global re-localization, place recognition, LiDAR SLAM, template matching, real-time performance

## Abstract

Place recognition is widely used to re-localize robots in pre-built point cloud maps for navigation. However, current place recognition methods can only be used to recognize previously visited places. Moreover, these methods are limited by the requirement of using the same types of sensors in the re-localization process and the process is time consuming. In this paper, a template-matching-based global re-localization framework is proposed to address these challenges. The proposed framework includes an offline building stage and an online matching stage. In the offline stage, virtual LiDAR scans are densely resampled in the map and rotation-invariant descriptors can be extracted as templates. These templates are hierarchically clustered to build a template library. The map used to collect virtual LiDAR scans can be built either by the robot itself previously, or by other heterogeneous sensors. So, an important feature of the proposed framework is that it can be used in environments that have never been visited by the robot before. In the online stage, a cascade coarse-to-fine template matching method is proposed for efficient matching, considering both computational efficiency and accuracy. In the simulation with 100 K templates, the proposed framework achieves a 99% success rate and around 11 Hz matching speed when the re-localization error threshold is 1.0 m. In the validation on The Newer College Dataset with 40 K templates, it achieves a 94.67% success rate and around 7 Hz matching speed when the re-localization error threshold is 1.0 m. All the results show that the proposed framework has high accuracy, excellent efficiency, and the capability to achieve global re-localization in heterogeneous maps.

## 1. Introduction

Simultaneous localization and mapping (SLAM) has been a hot research field in the past years. This technology enables automated guided vehicles (AGVs) to be applied in various applications, such as disinfection, security inspection, smart factories, etc. With the development of 3D LiDAR sensors and their affordable costs, 3D LiDAR-based SLAM has recently attracted more attention due to its long detection distance and insensitivity to illumination. Furthermore, how to re-localize a 3D LiDAR-based navigation system within a pre-built point cloud map is a classic but important problem in robotic applications.

The re-localization problem is usually regarded as a place recognition problem. Place recognition aims to recognize a previously visited place and can be used for various robot missions [1] such as loop detection in simultaneous localization and mapping (SLAM) [2], global localization for navigation [3], localization error recovery for a kidnapped robot [4], or multi-robot mapping [5]. The proposed re-localization framework focuses on the problem of re-localizing a robot in a pre-built map, and can be used for applications including, but not limited to, navigation and localization error recovery.

To fulfill re-localization in a pre-built 3D map, some researchers tried to adopt local descriptors in 3D point clouds [6,7]. Others tried to design global representative descriptors [2,8] for better computational efficiency and improved robustness. Applying deep learning technologies for feature representations [9,10,11] and similarity modeling [12,13] is a current hot trend in research. Also, some traditional techniques in image processing are extended for similarity modeling, such as chamfer matching and template matching [14]. Among these methods, global descriptor-based methods are robust and easy to be applied. However, these methods rely on comparing the similarity of the current scene with previously visited scenes, and therefore, have limitations when dealing with unvisited places.

In order to re-localize a robot globally with promising efficiency and accuracy, a real-time global re-localization framework is proposed. It is essentially a matching process of global descriptors and has two stages, namely, the offline building stage and the online matching stage. To overcome the limitation of dealing with unvisited places, virtual LiDAR scans in the whole map are first densely collected to build a template library offline, where the map can be built either by the robot itself previously, or by other 3D reconstruction techniques. Then, the robot can re-localize itself in the map by matching the current scene with the template library online. Specifically, at the offline building stage, a 3D point cloud map is first built by a LiDAR SLAM mapping algorithm to obtain the environment model. Then, a template library is established by collecting virtual LiDAR scans in a physical simulation engine using the environment model and robot URDF model. Finally, templates are hierarchically clustered and used to build a nearest-neighbor search engine consisting of locality-sensitive hashing (LSH) and a k-dimensional tree (KD tree). At the online matching stage, a cascade coarse-to-fine online template matching method with high efficiency is proposed, which takes full advantage of the properties of descriptors, the hierarchical structure between templates, and the nearest-neighbor search engine.

The main contributions are summarized as follows:

1. The proposed framework is designed to re-localize a robot globally in a pre-built map for navigation, even at locations that have not been visited during mapping.
2. The proposed framework also works with heterogeneous 3D maps. The map does not have to be acquired with the same sensors or the same robot. Instead, it could be provided by an external service, which is a trend that is becoming increasingly popular in the future for applications like autonomous driving.
3. An efficient cascade coarse-to-fine template matching method is proposed for online matching and has a promising real-time performance on large-scale datasets.
4. The proposed global re-localization framework is validated on both our simulated dataset and a public dataset, where it shows promising accuracy and efficiency.

## 2. Related Works

Three-dimensional LiDAR-based place recognition methods try to discern places that an AGV has visited before. They can be divided into local descriptor-based methods, global descriptor-based methods, and deep learning-based methods.

Local descriptor-based methods adopt similar ideas to visual SLAM. Instead of extracting local invariant descriptors of keypoints in 2D images, these methods extract local descriptors of keypoints in 3D point cloud data to build a bag of words (BOW). As for 3D point cloud local descriptors, on the one hand, some are extended from their 2D versions, such as 3D-SIFT [6] and 3D-SURF [7]. On the other hand, others can be extended from existing well-designed point cloud descriptors. For example, point feature histograms (PFHs) [15] and fast point feature histograms (FPFHs) [16] explore the geometry information by comparing local surface normal differences. Signature of Histograms of Orientations (SHOT) [17] divides the space around a keypoint into several regions and collects the normal distribution histogram of each region to generate a descriptor. Guo et al. [18] enriched SHOT with intensity information and proposed a new probabilistic keypoint voting approach to fulfill place recognition. However, detecting distinctive keypoints with high repeatability is still a challenging problem [19]. Also, identifying local descriptors is usually a compute-intensive operation, requiring keypoint detection and lots of local geometric calculations.

Global descriptor-based methods reduce the computational complexity and improve the robustness by extracting the features from the whole point cloud [3]. Kim et al. [2] proposed an egocentric spatial descriptor named Scan Context (SC). The SC descriptor encodes a whole point cloud in a 3D LiDAR scan into a matrix using the height information of the point cloud. It has been shown that extracting only the highest points of a visible point cloud outperforms other existing global descriptors. Wang et al. [8] proposed Intensity Scan Context by replacing the height information of the point cloud with the intensity and improved the robustness of the descriptor. Compared with [2], Wang et al. [20] also encoded the height information to obtain LiDAR Iris images but achieved rotation invariance by applying the Fourier transform to the descriptors. Cop et al. [21] established a local reference frame to generate a rotationally invariant descriptor regardless of viewpoint changes. Other than directly computing the height and intensity properties of point clouds, some researchers also use the geometric primitive properties of global point clouds to represent the scene abstractly. Wohlkinger et al. [22] proposed the Ensemble of Shape Functions (ESFs), which describes distance, angle, and area distributions on the surface of the partial point cloud using a series of histograms. Wietrzykowski et al. [23] represented both the scene and global map by planar segments and used multiple triplets of planar segments to generate a localization probability distribution. Schaefer et al. [24] extracted pole landmarks from 3D LiDAR scans to represent the scene to improve localization robustness, as poles are common and not affected by seasonal changes in urban environments.

Since global descriptors are computed in the LiDAR sensor’s reference frame, most descriptors are not translation-invariant. So they can only be used for place recognition within keyframe data collected on the mapping trajectory for loop-closure detection and coarse re-localization. Mapping an unfamiliar environment has now become easy. Benefiting from the long detection range of LiDAR, a global 3D point cloud map can be generated from point cloud data collected at only a few locations. In this case, the AGV must re-localize itself where it has not visited. Thus, the lack of translation invariance limits the application of existing place recognition algorithms.

To date, deep learning techniques have been applied to solve the place recognition problem in many processes, including point cloud feature extraction, descriptor similarity evaluation, and even end-to-end solutions. A typical way to leverage deep learning techniques is to extract semantic features by segmentation and detection in point cloud data. This semantic information can be used for building graphs, and place recognition is then carried out by solving a graph matching problem [9,10]. In contrast, Jiang et al. [25] also used graph matching but relied only on plane semantic information. Yin et al. [12] proposed a semi-handcrafted deep learning framework that learns representations by LocNet and solves the place recognition problem as a similar modeling problem. Chen et al. [13] utilized a deep neural network exploiting different cues generated from LiDAR data for similarity computation. Uy et al. [26] extracted point cloud descriptors using deep learning, allowing end-to-end training and inference to extract the global descriptor from a given 3D point cloud. Kim et al. [4] used SC maps to train a CNN for long-term place recognition. And, Fu et al. [27] even proposed a novel method to localize the vehicle by extracting and comparing the spatially discriminative feature maps of the satellite image patch and the LiDAR scans using a neural network. Although deep learning has surprised us in some datasets, deep learning-based methods require a large amount of data and manual labels for parameter training.

Thus, to re-localize the navigation system in the map even if the location has not been visited during the mapping stage, we propose that virtual LiDAR scans in the whole map are first densely collected to build a template library. Then, a cascade coarse-to-fine online template matching method is designed for efficient template matching, balancing both efficiency and accuracy.

## 3. The Proposed Re-Localization Framework

**Figure 1:**
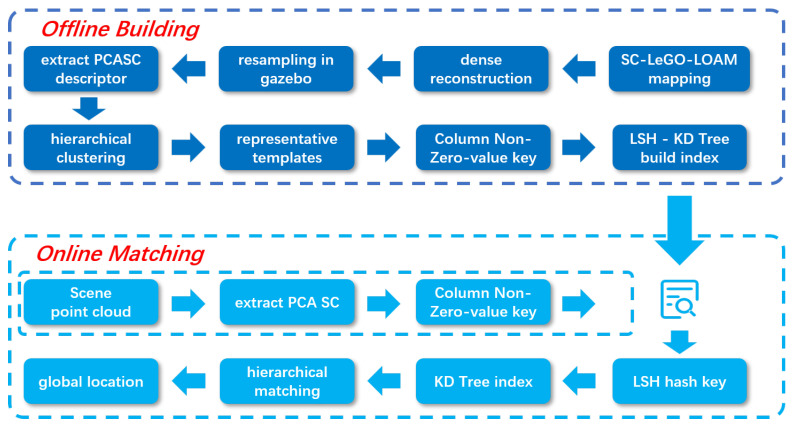
The proposed global re-localization framework.

As shown in Figure 1, the proposed global re-localization framework consists of an offline building stage, including environment modeling, virtual LiDAR scan collection, descriptor extraction, hierarchical clustering, nearest-neighbor index building, etc.; and an online matching stage, including descriptor extraction, coarse matching of template cluster candidates, and refined matching into clusters.

At the offline stage, a simulation environment is first established based on the 3D point cloud map. Then, an AGV model is placed in the simulation environment to collect point cloud data densely. After that, Principal Component Analysis Scan Context (PCASC) descriptors [28] can be extracted from each point cloud and stored as templates together with the metadata. All templates are clustered using the agglomerative hierarchical clustering method to produce representative templates of each cluster, which can reduce the overall number of templates and build a hierarchical structure. Finally, a nearest-neighbor search engine is built on representative templates. The construction of the template library involves template generation, template clustering, and organizing the templates by building an index, which is actually the entire offline build phase.

At the online matching stage, coarse matching based on the offline-built nearest-neighbor search engine is used to find template cluster candidates. Then, refined matching proceeds on these clusters for more accurate estimation results.

### 3.1. Template Generation

At present, dense reconstruction of unfamiliar environments has become easy using SLAM technologies and products like DJI Terra. A mesh model of the environment can be easily accessed and used to establish a simulation environment using the Gazebo physical simulation engine.

To collect point cloud data, an AGV model is placed at densely sampled positions in the Gazebo physical simulation engine while moving the AGV in small steps. At each sampling location, we detect collisions between the AGV and the environment, as in [29], which guarantees that the sampled templates make sense, i.e., the AGV will lie on the surface and not clash with any objects in the environment.

For each frame point cloud data point, a PCASC descriptor [28] can be extracted from it. The PCASC descriptor is an improvement of the Scan Context descriptor [2] to deal with the rotation invariant issue. It is achieved by performing a principal component analysis on the point cloud to determine a local reference coordinate before generating the SC descriptor. It speeds up the process of similarity computation between two descriptors by eliminating the ambiguity of rotation.

**Figure 2:**
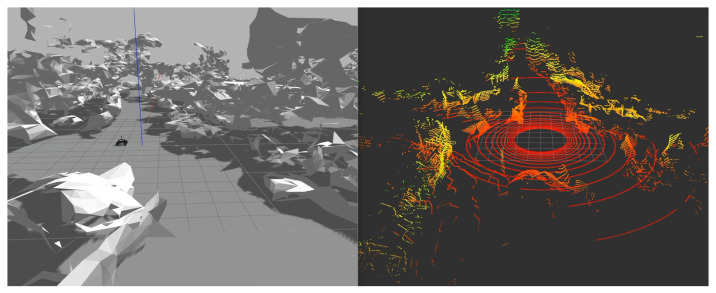
Resampling in Gazebo using mesh model. (**Left**) AGV in Gazebo with mesh model, collecting point cloud data. (**Right**) Collected point cloud data.

**Figure 3:**
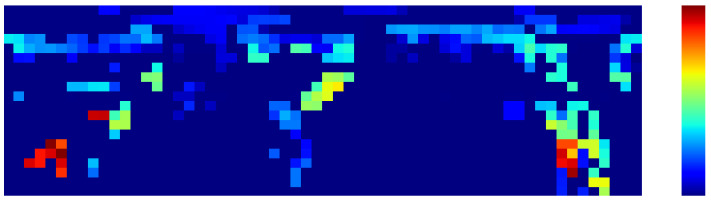
Extracted PCASC global descriptor (20 row × 60 column) from point cloud data in Figure 2.

At last, a template is defined as a collection of the PCASC descriptor and its metadata, where the metadata include the descriptor’s dimensions, robot’s position in the map, PCA angle, etc. The sampling procedure in the simulation engine and a visualized PCASC descriptor can be seen in Figure 2 and Figure 3.

### 3.2. Template Clustering

Since densely collected templates are oversampled, they are clustered by the agglomerative hierarchical clustering method to produce representative templates of each cluster. On one hand, the number of templates is reduced and representative templates are more distinguishable. On the other hand, a hierarchical structure can be established from the template library. At the online matching stage, the reduced templates can offer a coarse re-localization result efficiently, then the hierarchical structure can be used to conduct a hierarchical matching to improve the re-localization accuracy.

Templates are clustered in a bottom-up way, with each sample starting in its own cluster, and pairs of clusters are merged as one moves up the hierarchy. In order to decide which clusters should be combined, a measure of matrices of dissimilarity between clusters is required. The maximum, minimum, and average linkage matrices are widely used to specify the dissimilarity of sets as a function of the pairwise distances of samples in the cluster. And they can be formulated as

(1) $$D_{\max}\left(C_{i},C_{j}\right)=\max_{x\in C_{i},y\in C_{j}}d\left(x,y\right),$$

(2) $$D_{\min}\left(C_{i},C_{j}\right)=\min_{x\in C_{i},y\in C_{j}}d\left(x,y\right),$$

(3) $$D_{\mathrm{avg}\;}\left(C_{i},C_{j}\right)={\mathrm{avg}}_{x\in C_{i},y\in C_{j}}d\left(x,y\right),$$

where $D(C_{i},C_{j})$ is the distance matrix between clusters, *C* is a cluster of samples, *x* and *y* are samples belonging to each cluster, and d(x,y) is the distance matrix between two samples.

The distance matrix between two samples is calculated in the same way as in [28], which is

(4) $$d\left(x,y\right)=\frac{1}{n}\sum_{i=1}^{n}\left(1-\frac{x_{i}\cdot y_{i}}{∥x_{i}∥\cdot ∥y_{i}∥}\right).$$

As the similarity and distance are all normalized from 0 to 1, they can be converted by

(5) s(x,y)=1−d(x,y),

where s(x,y) is the similarity measure between two samples.

For point clouds collected at the same position, different sensor orientation leads to column vectors shifting of the descriptor but still expressing the same scene, which is also known as the viewpoint change challenge. So, in the similarity calculation of the SC descriptor, we calculate it as

(6) $$s_{\max}\left(x,y\right)=\max_{i\in n}\;\;s\left(x_{i},y\right),$$

where $s_{\max}\left(x,y\right)$ is the highest score by calculating the similarity between two descriptors by shifting *n* columns. Since the uncertainty of sensor orientation is eliminated through the principal component analysis of the point cloud, *n* is set to one when calculating the similarity between two PCASC descriptors in this paper.

Each cluster’s representative templates are selected by maximizing the total similarity between the representative template and all other templates within the cluster as

(7) $$t_{\mathrm{rep}\;}^{i}=\left\{x|\max_{y\in C_{i}}\sum s\left(x,y\right),x\in C_{i}\right\},$$

where $t_{\mathrm{rep}}^{i}$ is the representative template of cluster $C_{i}$.

The clustering procedure is based on similarity calculations between samples. The calculation has a complexity of $O(n^{2})$, where *n* is the total number of samples (templates). However, the computation cost is unaffordable when *n* increases, and samples far from each other may be clustered together, which is not preferred in building a local hierarchical structure. So, local constraints are introduced while clustering by only considering similarities of connected samples and regarding others as zero. Connectivity between samples is determined by searching the sample’s nearest neighbors. Local constraints significantly optimize the time for clustering.

### 3.3. Nearest-Neighbor Search Engine Building

The number of representative templates is much smaller after clustering than that of the original templates. Nevertheless, the total matching time still grows approximately linearly with the number of representative templates and the size of the PCASC descriptor. Therefore, a cascade matching is proposed to improve the real-time performance, consisting of coarse matching using the nearest-neighbor search engine to obtain template cluster candidates and refined matching on these clusters to improve accuracy.

The coarse matching aims to reduce the search scope by finding template cluster candidates. The coarse matching includes candidate searching and candidate sorting stages. The backbone of the coarse matching is a nearest-neighbor search engine combining locality-sensitive hashing (LSH) and k-dimensional trees (KD trees).

The search engine is built using column non-zero vectors (CNZ vectors) of all representative templates. The CNZ vector is a compression of the PCASC descriptor, a column vector with each element representing the number of non-zero values in each row of the PCASC descriptor. CNZ vectors are used to build the nearest-neighbor search engine to make the most of the sparsity of the PCASC descriptor. The CNZ vector remains rotation-invariant and compresses the number of dimensions of the data. For example, if the PCASC descriptor has 20 rows and 60 columns, we can obtain a CNZ vector of 20 dimensions, with each element representing the number of non-zero values in each row of the PCASC descriptor.

**Figure 4:**
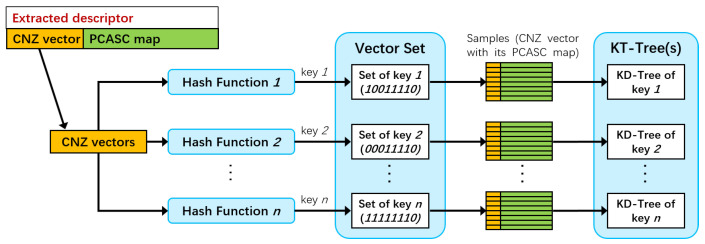
The nearest-neighbor search engine building process.

The nearest-neighbor search engine is built as in Figure 4. The CNZ vector is used to compute the hash key of the LSH and used as the key value to build the KD tree. CNZ vectors are first used to compute hash keys with appropriate hash functions, and similar vectors will produce the same hash keys and be grouped into a subset. Then, all samples in the subset are stored in a KD tree using CNZ vectors as keys. This can avoid exhaustive matching within the subset when querying online. LSH can determine a subset in an instant, and the KD tree of the subset can be used to efficiently search for nearest neighbors.

As for the hash function, we use the binary projection function, which projects the CNZ vectors onto several basis vectors and assigns a binary value (zero or one) according to the sign. Thus, the hash key is the sequence of all binary values (a string like 1101100110). LSH is probabilistic in finding nearest neighbors, so multiple hash functions are used to increase the success rate of finding nearest neighbors in this paper, as in [30]. In practice, we used the principal component analysis binary projection (PCA-BP) and random binary projection (RBP). The basis vectors of the PCA-BP function are obtained by principal component analysis of the CNZ vectors, while those of RBP are selected randomly.

### 3.4. Online Template Matching

**Figure 5:**
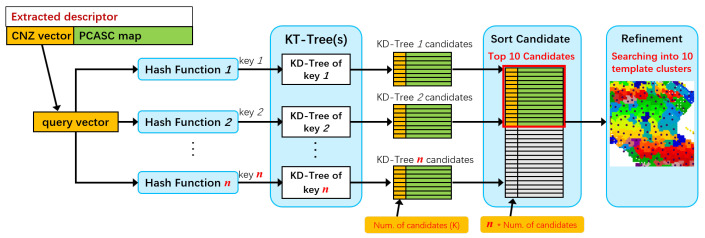
The online template matching procedure.

Online template matching consists of three stages, descriptor computation, coarse matching, and refined matching, as shown in Figure 5. The coarse matching aims to find template cluster candidates among all representative templates, and the refined matching aims to improve accuracy by searching these clusters.

At the descriptor computation stage, for each test sample noise points are filtered by applying a radius outlier removal filter on the LiDAR scan. Then, a PCASC descriptor is extracted from the point cloud as well as its CNZ vector.

At the coarse matching stage, template cluster candidates are determined by candidate searching and candidate sorting procedures. Firstly, the CNZ vector is used to calculate hash keys. Each hash key corresponds to a KD tree storing a set of similar templates, which produces K candidates. Distances are measured based on the Manhattan distance [31] between CNZ vectors during the candidate searching procedure. Secondly, all candidates are sorted together based on the distance definition, as in Equation (4). This candidate sorting procedure is based on the similarities between PCASC descriptors instead of the Manhattan distance between CNZ vectors, as this is more discriminative.

At the refined matching stage, the top 10 candidates are kept, and hierarchical searching is performed on the corresponding clusters for more accurate re-localization results.

In the candidate searching procedure of the coarse matching stage, CNZ vectors are taken as the key values to conduct the nearest-neighbor search. However, due to the similarity of some scenes and their CNZ vectors, wrong template cluster candidates may be found if *K* is too small, resulting in an increase in the final re-localization error. So it is critical to find the correct nearest neighbors using the CNZ vector for high re-location accuracy. The number of candidates *K* affects the re-location accuracy and calculation efficiency to a certain extent. It is a trade-off between matching speed and precision.

## 4. Experimental Results

In this section, the framework is firstly validated using simulated data. During the simulation, both templates and testing scenarios are collected using the same sensor in a simulation engine. A 3D point cloud map, built by adopting the SC-LeGO-LOAM [2,32] algorithm on the school campus, is used as the environment model. Then, the framework is further validated on The Newer College Dataset [33] using real LiDAR scans. During validation on the public dataset, templates are collected in the simulation engine using a pre-built 3D map collected by a Leica BLK360 sensor. Testing scenarios are real 3D LiDAR scans (point cloud) collected by Ouster 64 multi-beam LiDAR. Thus, the sensor used for mapping and that used for re-localization are heterogeneous in the public dataset used for validation.

In Section 4.1, we show the visualization results of hierarchical clustering and illustrate the importance of local constraints during similarity calculation in terms of saving total clustering time while not decreasing clustering performance. In Section 4.2, we demonstrate the feasibility of enabling global descriptors to re-localize globally in a 3D point cloud map by densely sampling descriptors offline. The results in Section 4.2 are obtained without any acceleration, and the only purpose is to show the global re-localization ability. Then, the accuracy and efficiency of the algorithm are validated using simulated data and real data, respectively, in Section 4.3 and Section 4.4. Some potential capabilities and limitations of this work are discussed in Section 5.

The experiments were carried out on a computer with an Intel 9700 CPU and 32 G DDR4 RAM. The code was developed in the Python language and the running time was calculated using a single thread.

### 4.1. Configuration of Clustering

**Figure 6:**
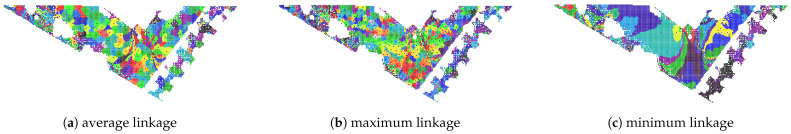
Different clustering principles while merging clusters using 10 K samples. Clusters are identified from each other by color, where each dot represents a real sample, and representative templates for each cluster are plotted with black dots.

First, we compare the performance of different clustering principles. The visualization results of clustering under different principles are shown in Figure 6. For clear visualization, only part of the whole map is shown using 10 K samples.

Maximum linkage constrains that the maximum distance between each pair of samples in the cluster is lower than the clustering threshold and produces the best local constraints. In contrast, average and minimum linkage loosen the constraints and prefer to link more dissimilar samples into one cluster. The minimum linkage criterion produces the worst clustering result.

Second, to prove that local constraints during the clustering procedure can distinctively decrease the total clustering time while not decreasing the matching accuracy, we repeat clustering and matching with and without local constraints. The statistical results in Table 1 are collected using 100 K samples, clustered by the maximum linkage principle. The success rate is defined as the percentage of test samples for which the re-localization error is less than the threshold.

We compare the clustering and matching performance with and without local constraints. The clustering and matching results are similar in the following aspects: the number of clusters, time for clustering, and re-localization errors. This shows the local connection constraints have no negative influence. In contrast, the time cost by similarity calculation when not using local constraints is 2000 times that with constraints. As observed in Table 1, the number of clusters without constraints is a little smaller, and the success rate is a little higher because similar samples are clustered together despite how far they are apart from each other.

All things considered, using local constraints during similarity calculation is important for saving total clustering time while not decreasing clustering performance.

### 4.2. Global Re-Localization Performance

To prove the global re-localization ability of the proposed framework, the PCASC descriptors of trajectory points and those of resampled points are used to carry out a cross-matching. Both of them are collected in the simulation engine using a 3D point cloud map built by the SC-LeGO-LOAM [2,32] algorithm in the school campus. The trajectory points mean the PCASC is collected where the AGV has visited while mapping, while the resampled points are collected in the whole map.

During the test, 100 K samples are divided into 50 K template samples and 50 K test samples when using resampled templates to match the resampled template scenes.

The results in this subsection are obtained without any acceleration, and the re-localization is achieved only by exhaustive matching. The purpose of this experiment is to show the global re-localization capability of the proposed framework. The matching time is the average time to match a scene using the Python implementation and is almost linearly related to the number of templates.

The statistical results in Table 2 show that if the template library is built from trajectory points and tested by resampled points, the success rate is extremely low. Because the trajectory points cannot cover the entire map, it only succeeds if the test sample is within a limited distance of the trajectory path. In contrast, if the template library is built from resampled points, all test samples can be matched successfully, whether tested by resampled points or by trajectory points.

The results indicate that global descriptors like PCASC have a limitation when used for global re-localization because of the lack of translation invariance. And it is feasible to re-localize a robot using global descriptors with the proposed framework.

### 4.3. Validation on Simulated Data

In this subsection, we test the performance of the framework using 100 K templates collected through the Gazebo physical simulation engine and 2000 test samples randomly selected from the templates. Each sample is matched with the template library to obtain the re-localization result.

**Figure 7:**
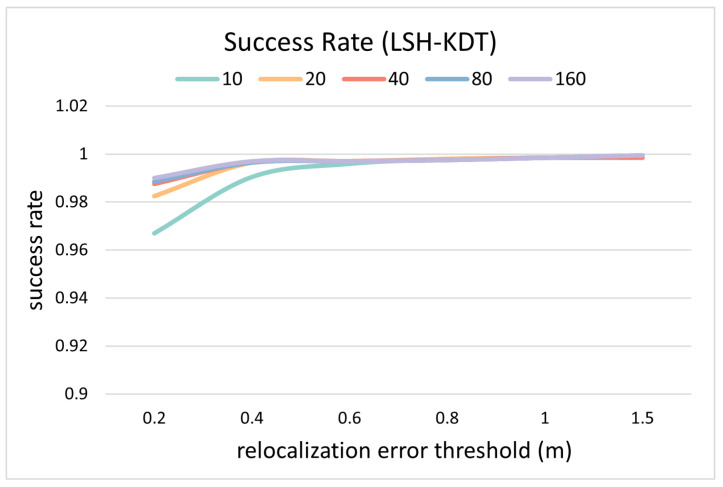
Accuracy comparison between different number of candidates on simulated data.

The distance between the re-localization result and the reference position is counted as the re-localization error in Figure 7. The success rate curve is defined as the proportion of the test samples whose re-location error is less than the current threshold. Each curve represents a matching success rate curve for a certain *K* value, where *K* indicates the number of template candidates.

**Figure 8:**
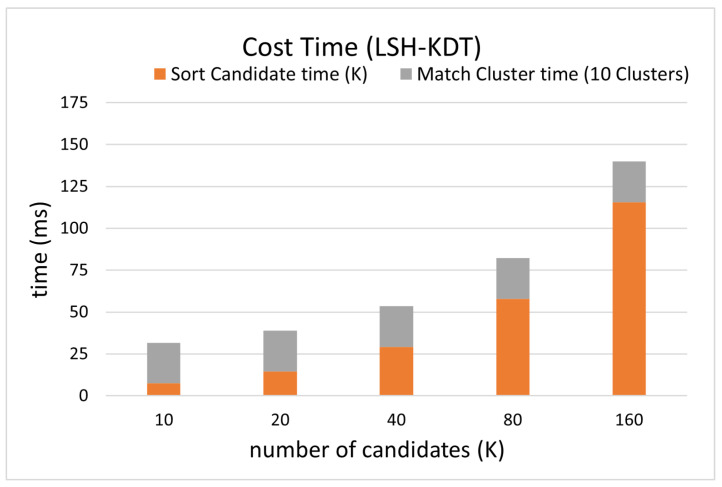
Efficiency comparison between different numbers of candidates on simulated data.

The online matching is divided into four processes including descriptor generation, template cluster candidate searching, candidate sorting, and hierarchical searching in the clusters, of which the running time of the last two are counted in Figure 8.

Since the test sample is a subset of templates, the success rate at 0.2 m should ideally be 100%. But the success rate is less than 100%, and it increases as the number of candidates increases. This indicates that CNZ vectors from similar environments reduce the search accuracy of the nearest neighbor, thus reducing the re-localization accuracy. This problem is alleviated by increasing the number of candidates (*K*) at the cost of reducing efficiency.

When the cluster threshold is 0.1, the number of template candidates is 10, then the average time for matching is about 30 ms, as in Figure 8, with a success rate of 99.85% at the re-localization threshold of 1.0 m, as in Figure 7. Considering the time for calculating one PCASC descriptor is about 60 ms, the re-localization speed is about 11 Hz overall.

### 4.4. Validation on Public Dataset

The proposed re-localization framework is further validated on a public dataset with real LiDAR scans and ground truth robot position in this subsection. The dataset used in this subsection is The Newer College Dataset [33], which satisfies the scenario that an AGV can map the whole environment by visiting only a small path but can re-localize itself within the whole area.

**Figure 9:**
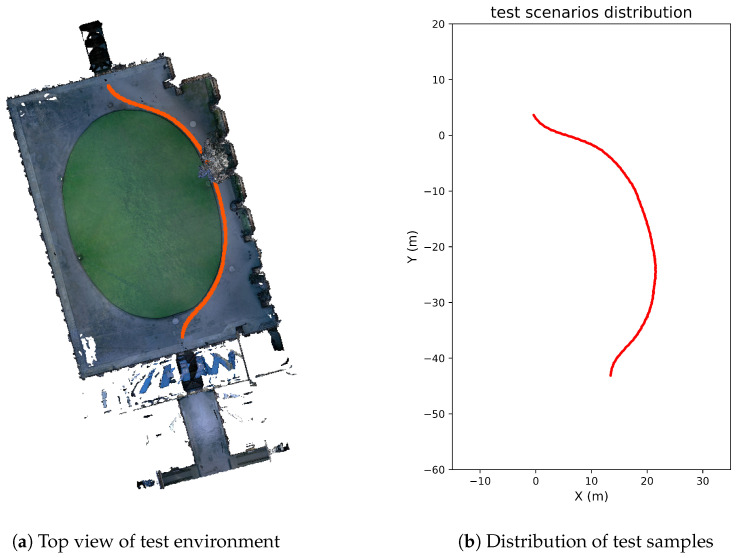
The NCD dataset used for validation. (**a**) Top view of the test environment. Each test sample is plotted with a red dot at the location in the environment where it was collected. (**b**) Distribution of test samples on X-Y plane.

The quad in the dataset is about 2000 square meters, as shown in Figure 9. A reconstructed mesh model based on a point cloud obtained from a Leica BLK360 sensor is used to collect 43,770 templates, using a step size of 0.2 m. The total size of the templates is 679 MB. Then, 600 real LiDAR scans obtained from an Ouster OS-0 sensor are used as test samples for querying. And, 43,770 resampled virtual LiDAR scans are used as a template library for matching.

**Figure 10:**
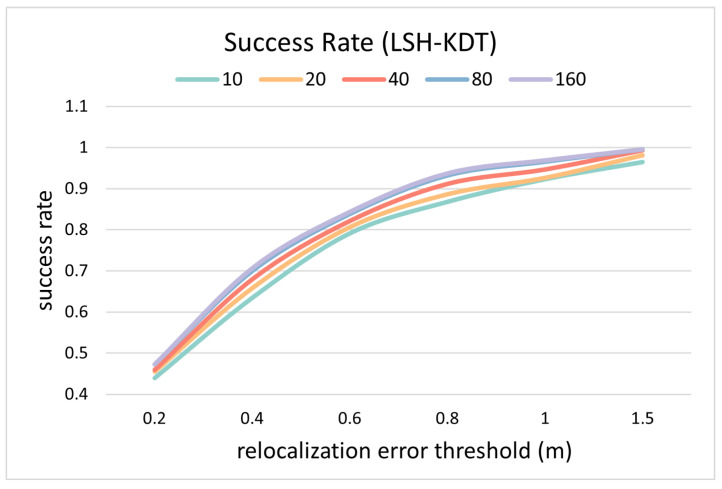
Accuracy comparison between different numbers of candidates on real data.

**Figure 11:**
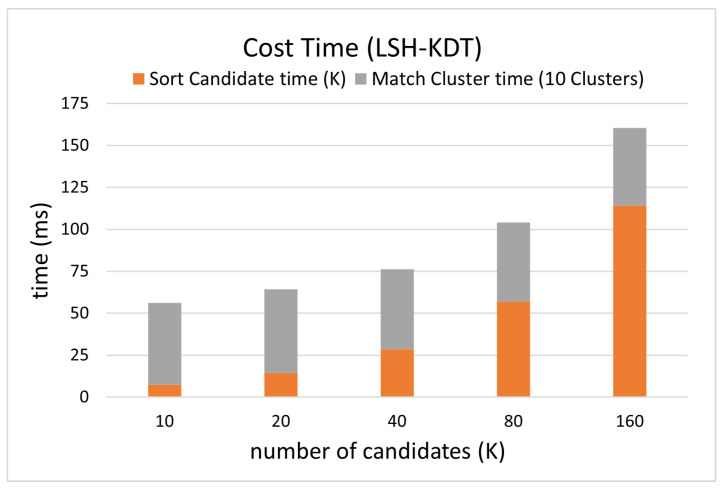
Efficiency comparison between different numbers of candidates on real data.

The accuracy and efficiency change as the number of template candidates increases, as calculated in Figure 10 and Figure 11. The definition is the same as in Figure 7 and Figure 8.

In terms of efficiency, the time for matching 10 clusters in our dataset is 30 ms but in the NCD dataset is about 56 ms. This is caused by the difference in the average number of samples per cluster. More specifically, in the template clustering phase, all samples are recursively merged until the clustering threshold is reached. The clustering threshold can be the maximum similarity difference between samples in the cluster (used in this paper) or the maximum number of samples contained in the cluster. When different environmental models are used, due to the characteristics of the environment model, the number of samples contained in the cluster may be different even if the clustering threshold is the same. For example, the average number of samples per cluster in the NCD dataset is about twice that of our dataset while using the same clustering threshold of 0.1, as is the ratio of the matching time between them.

In terms of accuracy, it can be seen from Figure 10 that the matching accuracy on the public dataset has decreased compared to the tests on the simulated dataset in Figure 7. Specifically, the success rate in Figure 10 is lower than that in Figure 7 when the same localization threshold is chosen. This is because, although the point clouds were acquired for the same environment, there are still differences between the virtual and real point clouds. For this reason, the matching result may not be exactly the same as the ground true value, resulting in a decrease in accuracy. However, if an acceptable threshold is chosen, such as specifying a maximum localization error of 1.0 m, 94.67% of the test samples can still be successfully matched, which can be seen from Figure 10.

**Figure 12:**
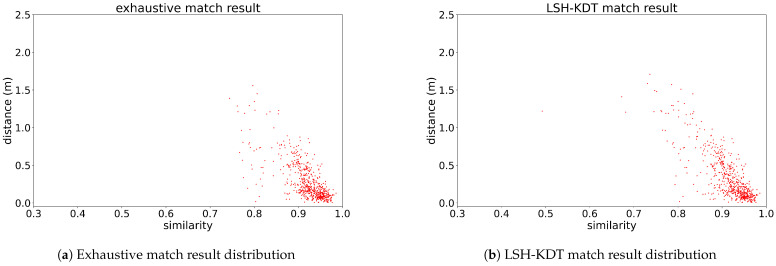
Match result distribution between distance and similarity. (**a**) Exhaustive match result distribution. (**b**) LSH-KDT match result distribution.

The matching result distribution of this test is plotted in Figure 12, with each point representing a match result for one test sample. The horizontal axis indicates the similarity between the test sample and the matched template. And the vertical axis indicates the Euclidean distance between the ground truth position of the test sample and the coordinates of the matched template, which is called the matching error or localization error in this paper. Figure 12a shows the result of the exhaustive matching method and Figure 12b shows the result of the proposed cascade coarse-to-fine online template matching method. For the exhaustive matching result in Figure 12a, the success rate is 97.83% when the threshold is set to 1.0 m, taking approximately 191 s per sample. For the proposed LSH-KDT match result in Figure 12b (*K* is 40), the success rate is 94.67% when the threshold is set to 1.0 m, taking approximately 0.135 s matching per sample. Figure 12 is used to illustrate that the degradation in matching accuracy is not a defect in the proposed framework, but a result of the inconsistency of the point cloud data. It also shows that the proposed framework achieves almost the same accuracy performance as exhaustive matching.

Since the exhaustive match is a traversing matching process without acceleration, it stands for the best performance under the experimental setup. But the re-localization errors of some test samples are still bigger than 0.2 m. This might be caused by two factors. One is the difference in noise distribution between real LiDAR scans and templates resampled by the Gazebo physical simulation engine. The other is the similarity between scenarios. Under this premise, the LSH-KDT match result achieves good performance compared to the exhaustive match result. The slight difference in their matching accuracy is also caused by the error of searching for the nearest neighbor using CNZ vectors of similar scenarios, as in Figure 7.

## 5. Discussion

**Figure 13:**
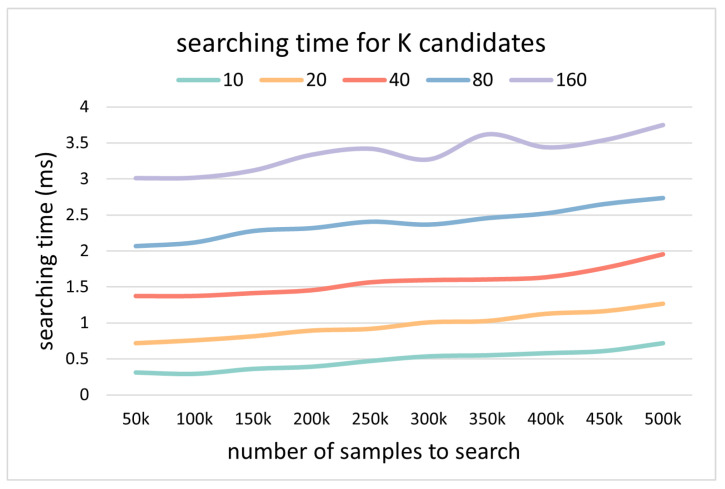
The change in candidate searching time with the increase in the number of representative templates for different *K* values.

The online matching process is divided into four parts, starting from descriptor generation and ending with receiving an estimated position, which are descriptor generation, candidate searching, candidate sorting, and hierarchical searching into clusters. The descriptor generation time is constant, about 60 ms. The candidate searching time is less than 1 ms during testing, so it is too small to be counted in Figure 8 and Figure 11. The candidate searching time varies with the number of samples but is negligible, as shown in Figure 13, which shows the potential to use the framework in a large-scale environment. The candidate sorting time is directly proportional to the number of candidates, resulting in a higher success rate and some performance loss costs. Finally, the matching time of the cluster is only related to the number of samples contained in the cluster. In conclusion, the proposed matching framework can guarantee similar matching efficiency even on a much bigger template library and larger-scale environment.

It is noted that in the validation phase on the NCD dataset, the LiDAR scans collected by the real robot are incomplete because the LiDAR’s field of view is partly blocked by the operator. From the point cloud data perspective, this results in some sectors being completely blank. From the point of view of the PCASC feature map, this will lead to about three all-zero column vectors, failing similarity calculation. Therefore, we complete the missing part of the real point cloud using virtual point clouds rendered at the same position and orientation.

## 6. Conclusions

This study proposed a template matching-based re-localization framework to re-localize a robot navigation system globally in a pre-built 3D point cloud map in real time, even at unvisited places where the robot has not actually visited before. The global re-localization ability and accuracy are achieved by resampling virtual LiDAR scans in the whole map when building the template library. The real-time matching efficiency is achieved by template clustering and the proposed cascade coarse-to-fine online template matching method. The proposed global re-localization framework is tested with a pure python implementation using one thread on both simulation data and a public dataset. In the simulation with 100 K templates, the proposed framework achieves a 99% success rate and around 11 Hz matching speed when the re-localization error threshold is 1.0 m. In the validation on The Newer College Dataset with 40 K templates, it achieves a 94.67% success rate and around 7 Hz matching speed when the re-localization error threshold is 1.0 m. Thus, the proposed global re-localization framework is both effective and efficient.

Moreover, the framework’s efficiency can be greatly improved when optimized using the C++ language, SIMD instruction, multiprocessing coding, etc., which is predictable.

## Figures and Tables

**Table 1:** Hierarchical clustering with or without local constraints.

Number of Templates	Local Constraint	Similarity Compute Time (s)	Cluster Threshold	Number of Clusters	Success Rate at Re-Localization Error Threshold (m)				
					0.2	0.4	0.6	0.8	1.0
100 K	None	228 s × 100 K	0.2	15 K	99.99%	100%	100%	100%	100%
			0.3	7.7 K	99.99%	99.99%	99.99%	99.99%	99.99%
			0.4	4.4 K	99.93%	99.95%	99.96%	99.97%	99.97%
			0.5	2.4 K	99.72%	99.79%	99.84%	99.88%	99.89%
	Using KNN	0.11 s × 100 K	0.2	15 K	99.99%	100%	100%	100%	100%
			0.3	8.4 K	99.99%	99.99%	99.99%	99.99%	99.99%
			0.4	6.0 K	99.77%	99.88%	99.92%	99.93%	99.94%
			0.5	4.6 K	98.80%	99.30%	99.61%	99.69%	99.74%

**Table 2:** Ability to re-localize globally (without any acceleration).

Query Scene	Number of Scenes	Template Library	Number of Templates	Match Time (s)	Success Rate at Re-Localization Error Threshold (m)				
					0.2	0.4	0.6	0.8	1.0
Resampled points	100 K	Trajectory points	1.6 K	2.3	2.39%	5.02%	7.78%	10.07%	12.51%
Resampled points	50 K	Resampled points	50 K	66.3	39.70%	96.15%	98.19%	98.72%	99.05%
Trajectory points	1.6 K	Resampled points	100 K	139.3	100%	100%	100%	100%	100%

## Data Availability

The original data presented in the study are openly available in The Newer College Dataset at https://ori-drs.github.io/newer-college-dataset/.
